# Intelligence

**Published:** 1829-10

**Authors:** 


### INTELLIGENCE.

### MONTHLY REPORT OF DISEASES.

We stated in our last report that the cases of Cholera which had fallen under our own observation had been mild, and easily controlled by medicine. In the course of the last fortnight, we have seen three cases of this disease of unusual severity, although the patients ultimately recovered. In each the attack was very sudden; the vomiting and purging violent and incessant. A state of extreme and alarming exhaustion very rapidly supervened. The surface of the body became cold; the pulse was remarkably slow, in two of the cases under fifty, and there were severe cramps in the legs. Almost immediate relief was obtained in each of these cases from hot brandy and water, after which opium was administered, and mild diluents freely given. The patients quickly recovered. If, in compliance with the Broussaian doctrines, stimuli had been withheld in these cases because there were great irritability of the stomach,* there can, we think, be no doubt that they would have terminated fatally.

* Propositions de Médecine, par F. J. V. Broussais, ccxi.

---

*Surgical Lectures.* Mr. Howship, surgeon to the St. George's Infirmary, &c. will commence, on the 5th instant, a course of surgical lectures. These lectures, throughout each course, will be copiously illustrated by the notes of cases or dissections that have fallen under Mr. H.'s personal observation. The appearances before and after death will be exhibited in an extensive series of drawings, and also by a numerous and choice collection of morbid anatomical preparations, many of them finely injected. Thus will be especially demonstrated all the peculiarities in the morbid anatomy of hernia, aneurism, injuries of the head and spine, urinary calculi, diseases of the kidneys, bladder, prostate gland and urethra; stricture; abscess or disease, common and specific, in every part of the alimentary canal. Numerous specimens of various diseases in the bones, with drawings illustrative of the minute appearances and actual condition of each morbid structure, as ascertained and demonstrated by the solar microscope, are also prepared, to facilitate the progress of the student.

---

*Midwifery.* Mr. Jewel, surgeon accoucheur to the Middlesex Infirmary, &c. commences his next course of lectures on Midwifery, and the various diseases connected with that branch of medical science, on Friday, October 2d, at the Theatre of Anatomy, Little Windmill street. To the first case of labour the student is accompanied by Mr. Jewel, or an experienced pupil; and, in all protracted and difficult cases, Mr. J. attends for the purpose of giving clinical instruction.

---

*Medical and General Botany.* Mr. Gilbert Burnett commences his course on Tuesday, 13th October, at seven o'clock in the evening, at the Theatre of Anatomy, Great Windmill street.

---

### MONTHLY LIST OF MEDICAL BOOKS.

### METEOROLOGICAL JOURNAL,

By Messrs. HARRIS and Co. Mathematical Instrument Makers, 50, High Holborn.

| August | Moon | Rain gauge. | Thermom. | | | Barometer. | | De Luc's Hygrom. | | Winds. | | Atmospheric Variations. | | |
|---|---|---|---|---|---|---|---|---|---|---|---|---|---|---|
| | | | 9 a.m. | 2 p.m. | 10 p.m. | 9 A.M. | 10 P.M. | 9 A.M. | 10 P.M. | 9 A.M. | 10 P.M. | 9 a.m. | 2 p.m. | 10 p.m. |
| 20 | | | 57 | 63 | 50 | 29.12 | 29.51 | 56 | 55 | WSW | NW | Show'ry | Show'ry | Show'ry |
| 21 | ☾ | .15 | 60 | 64 | 57 | .69 | .82 | 53 | 50 | WNW | WNW | Fine | Fine | Fine |
| 22 | | | 60 | 65 | 54 | .79 | .58 | 50 | 54 | SW | SS W | Cloudy | Fine | Rain |
| 23 | | | 57 | 67 | 57 | .42 | .29 | 55 | 60 | W | WNW | Rain | Rain | Cloudy |
| 24 | | | 59 | 62 | 50 | .17 | .53 | 62 | 53 | NWv. | WSW | Cloudy | Show'ry | Cloudy |
| 25 | | .79 | 57 | 64 | 50 | .76 | .94 | 50 | 50 | WNW | Wvar. | Fine | Fine | Fine |
| 26 | | | 59 | 64 | 58 | .94 | .60 | 55 | 56 | SW | S | Cloudy | Cloudy | Cloudy |
| 27 | | | 62 | 65 | 53 | .50 | .34 | 56 | 55 | W | SW | Fine | Show'ry | Rain |
| 28 | | .106 | 58 | 61 | 54 | .40 | .60 | 55 | 58 | NWv. | NW v. | Show'ry | Show'ry | Fine |
| 29 | ● | .15 | 58 | 64 | 52 | .80 | 30.00 | 60 | 56 | NE v. | NNE | Cloudy | Fine | Fine |
| 30 | | | 56 | 62 | 53 | 30.03 | 30.00 | 56 | 57 | NE | NE | Fine | Fine | Fine |
| 31 | | | 58 | 58 | 55 | 29.97 | 29.93 | 57 | 60 | NE | NE | Cloudy | Fine | Cloudy |
| Sept. | | | | | | | | | | | | | | |
| 1 | | | 56 | 62 | 51 | .86 | .83 | 60 | 60 | NE | N | Cloudy | Cloudy | Cloudy |
| 2 | | .05 | 60 | 66 | 53 | .81 | .89 | 60 | 54 | NNW | NNW | Cloudy | Fine | Fine |
| 3 | | | 58 | 63 | 54 | 30.04 | .99 | 54 | 52 | NNE | WSW | Fine | Fine | Fine |
| 4 | | | 60 | 62 | 53 | 29.89 | .77 | 52 | 52 | WSW | WNW | — | — | — |
| 5 | | | 60 | 64 | 53 | .60 | .37 | 52 | 53 | S | S | Cloudy | Fine | Cloudy |
| 6 | ☽ | | 62 | 67 | 52 | .41 | .48 | 53 | 54 | SSW | SW | Fine | Fine | Cloudy |
| 7 | | | 58 | 65 | 50 | .53 | .45 | 54 | 55 | WSW | S | Fine | Show'ry | Rain |
| 8 | | | 56 | 64 | 52 | .45 | .47 | 58 | 58 | S var. | SW | Cloudy | Show'ry | Fine |
| 9 | | .55 | 60 | 65 | 57 | .52 | .56 | 56 | 59 | WSW | S | Fine | Fine | Rain |
| 10 | | .27 | 62 | 67 | 55 | .31 | .17 | 61 | 56 | S | WSW | Rain | Fine | Rain |
| 11 | | .11 | 60 | 65 | 48 | .42 | .49 | 52 | 55 | WNW | WSW | Cloudy | Cloudy | Rain |
| 12 | | .34 | 59 | 62 | 54 | .54 | .50 | 57 | 55 | WSW | W | Fine | Fine | Fine |
| 13 | ○ | | 58 | 62 | 48 | .40 | .18 | 55 | 57 | SW | WSW | Fine | Fine | Rain |
| 14 | | .63 | 54 | 60 | 46 | .09 | .26 | 57 | 55 | WNW | WSW | Fine | Fine | Cloudy |
| 15 | | | 52 | 59 | 50 | .52 | .72 | 55 | 61 | WSW | W | Fine | Fine | Fine |
| 16 | | | 53 | 56 | 46 | .45 | .60 | 67 | 59 | ENE | NE | Rain | Rain | Fine |
| 17 | | | 52 | 57 | 50 | .83 | .61 | 57 | 67 | W | SW | Fine | Cloudy | Cloudy |
| 18 | | .40 | 55 | 59 | 51 | .09 | .12 | 67 | 65 | S | W | Cloudy | Cloudy | Cloudy |
| 19 | ☾ | | 54 | 55 | 50 | .34 | .63 | 65 | 62 | NWv. | N | Rain | Rain | Fine |

The quantity of Rain fallen in the month of August, was 4 inches and 45-100ths.

